# Psychological and Pedagogical Support for Parents of Children with Special Educational Needs

**DOI:** 10.5334/cie.103

**Published:** 2024-04-16

**Authors:** Zauresh Issabayeva, Aizhan Sapargaliyeva, Galiya Shubayeva, Kuanysh Shalabayeva, Gulnur Ismagambetova

**Affiliations:** 1 Higher School of Pedagogy and Psychology, Zhetysu University, Taldykorgan, Republic of Kazakhstan; 2 School of Transformative Humanities, Almaty Management University, Almaty, Republic of Kazakhstan; 3 Department of Special Pedagogy, Kazakh National Women’s Teacher Training University, Almaty, Republic of Kazakhstan; 4 Higher School of Physical Culture and Art, Zhetysu University, Taldykorgan, Republic of Kazakhstan; 5 Department of Theoretical and Practical Psychology, Kazakh National Women’s Teacher Training University, Almaty, Republic of Kazakhstan

**Keywords:** fairy tale activity, learning models, personal competencies, family relationships, hyper protection

## Abstract

This study primarily aimed to develop a program that aids parents of children with Special Educational Needs (SEN), placing emphasis on the challenges encountered in communication and understanding. The Eidemiller and Justizkis’s (2008) Family Relationship Analysis (FRA) served as the core method of the research. As inclusive education progresses, new challenges incessantly emerge necessitating immediate effective solutions. This research spotlights the triad of teachers, psychologists, and parents. A cornerstone of this research is the emphasis on nurturing a unique bond between school psychologists or teachers and parents. This bond should be anchored in mutual trust, motivation, and a conducive psychological environment. The overarching goal is to boost parental motivation, alleviate concerns linked to the child’s condition, reinforce family relationships, and cultivate a positive enabling environment for the child. The study culminated in the rollout of a tailored program connecting parents with educational professionals. This program encompassed lectures and exercises delving into understanding challenges, coupled with innovative methods like fairy tale activities and initiatives to foster tolerance. Feedback indicated a notable positive impact on parents. The research underscores the necessity of fostering parental confidence and assuaging fears regarding their child’s condition. In this context, the influence of peers and friends on the development of inclusive education should be considered in future studies.

The implementation of inclusive education is one of the main approaches to solve problems in the education system, providing those future teachers as well as psychologists, working with SEN children, are trained in a new way, and separate educational programs are created (Selvachandran et al., 2022). SEN children require additional support due to physical, cognitive, emotional, or behavioural challenges in attaining their learning potential (Law of the Republic, 2021). Therefore, it is necessary to equip parents with pedagogical and psychological skills as the parents’ participation in educational work and in communication with the school proves a major problem (Byhar et al., 2021). Lazarenko and Kiseleva (2020) describe the problems and conditions of inclusive education for children with musculoskeletal disorders, where the main focus is the availability of the necessary technologies, benefits and space for children with such diseases.

According to the Republic of Kazakhstan’s Ministry of Education (2021), the number of SEN children rose by at least 55 thousand. The increase in the number of SEN children could be attributed to several factors. Firstly, the adoption of a new normative act according to which all conditions are created (including new curricula and manuals) so that SEN children have the opportunity to study on an equal footing with other children. Secondly, enhanced diagnostic capabilities and greater awareness of developmental and learning disabilities might have led to better identification. Thirdly, social acceptance has also made parents more proactive in seeking evaluations for their children. Fourthly, several factors could have contributed, including changes in environmental exposures, broader definitions of certain conditions, better access to educational and health services, and socio-economic challenges. Lastly, population dynamics, such as migration, could have introduced new patterns in the demographics (Tkachenko, 2021; Chechko et al., 2022).

Consequently, it is very important that teachers and psychologists accompany parents to help them work with their children. It should be noted that the SEN category, according to the regulations, includes not only children with special education needs, but also with difficult economic or social status (Law of the Republic, 2021). Since 2011, inclusive education has been gradually developing in Kazakhstan. Inclusive education is an approach that accommodates all children, regardless of their conditions, ensuring equal access and eliminating barriers to learning (Law of the Republic, 2021).

Kabdualiyeva and Zubakova (2021) based on data from psychologists Mairamyan and Agavelyan, conducted an analysis of the phases parents endure whilst accompanying SEN children. At the first stage there was shock and also difficulties in comprehending the child’s peculiarities followed by an awareness which eventually included all family members (Vovchenko, 2021). It should be noted that parents are the engine that start the process of children’s adaptation to the environment, and also contribute to improved learning (Dor, 2022). Alisauskiene and Kairiene’s (2016) research, concluded that a joint interaction of teachers and parents proves the most difficult process in inclusive education, but also gives a better result among SEN children.

In the Republic of Kazakhstan, the approach to teaching SEN children has evolved over the years reflecting the global shift towards inclusive education. Traditionally, SEN children were primarily taught in specialized institutions, isolated from their peers. However, with the realization of the numerous benefits of inclusive education, there has been a concerted effort to integrate SEN children into mainstream classrooms. As of the current educational framework in Kazakhstan, wherever feasible, SEN children are predominately included in regular classes. This integration operates on the belief that all children, regardless of their physical, emotional, or cognitive challenges, have the right to be educated alongside their peers in a nurturing, inclusive environment. Such an approach not only benefits SEN children through enhanced social interactions and peer learning, but also fosters empathy and understanding among typical developing children (Zhasulan et al., 2022).

To support this inclusion the Kazakhstani educational system has taken several measures. Firstly, schools have been equipped with resources and infrastructure changes, such as ramps for wheelchair users, tactile pathways for visually impaired children, and soundproof rooms for those with auditory sensitivities. These infrastructural developments ensure physical inclusivity. Secondly, training programs for teachers have been implemented to equip them with the necessary skills to address the diverse needs of all children. These programs focus on differentiated instruction, adaptive teaching methodologies, and the use of assistive technologies. Thirdly, in schools with a higher concentration of SEN children, special education professionals are present to provide additional support.

Furthermore, though the philosophy of inclusion is gaining momentum there are pockets of resistance among educators and parents alike stemming from a lack of understanding or preconceived notions about disabilities (Mukashev & Somerton, 2023). Based on the analysis provided it can be concluded that for children with SEN the roles of both teachers and psychologists are crucial. Nevertheless, the primary guidance from parents is equally important for their development (Attard & Booth, 2023; Curra et al., 2023).

## Method

This study is theoretical and empirical in nature and aims to identify the main pedagogical and psychological conditions for supporting parents’ relationships with SEN children. A program was created to accompany parents with teachers and psychologists who deal with SEN children (Table 1).

**Table 1:** Program for accompanying parents with SEN children.


DAY	THE ESSENCE OF THE PROGRAM	AMOUNT OF TIME	FEEDBACK MECHANISM	EXPECTED OUTCOME

1	acquaintance with parents; questionnaire on how well parents know the needs of their children; methodology by E.G. Eidemiller and V. Justizkis; discussion of the results of the day.	2-3 hours	Questionnaire, open discussion, individual reflection	Establish trust and open communication lines. Understand parental baseline knowledge. Clarity on family relationships.

2	short lecture “Understanding”; exercise “Matches”; exercise “Creativity”; exercise “Action”; discussion of the results.	Lecture – 1 hour Each exercise for 20 minutes	Interactive poll, feedback forms, group discussion	Enhanced mutual understanding. Improved communication strategies. Recap and reinforce learning.

3	lecture “Emotions”; discussion of the results.	Lecture – 1 hour Exercise – 10 minutes	Role play feedback, feedback sheets	Improved emotional intelligence. Confirmation of grasped concepts.

4	exercise “Attention”; exercise “Activity”; discussion of problems.	Exercise – 40 minutes	Pair-sharing feedback, Group reflections	Enhanced focus on children’s needs. Engage in proactive measures for child’s growth.

5	method “Fairy tale activity”; lecture “Dialogue or monologue”; discussion of the results.	Fairy tale activity – 30 minutes Lecture – 1 hour	Story analysis feedback, role play feedback	Foster creativity and mutual understanding. Improve conversational skills.

6	methodology of communicative tolerance; exercise “Sculptures”; discussion of the results.	Method of communicative tolerance – 40 minutes Exercise – 15 minutes	Group consensus feedback	Foster understanding and tolerance. Engage in non-verbal communication

7	discussion of the results of the program; highlighting the positive/negative aspects of the program; final conversation.	2 hours	Group feedback session, individual feedback forms	Comprehensive understanding of the program. Recognize areas of improvement.


### Participants

The participants in this research were educators, social scientists, and parents of SEN children. Of the 50 Kazakh parents surveyed, 38 were women and 12 were men, predominantly aged between 30 and 50. Most children were enrolled in primary (60%) education and secondary (30%) education, with the remaining 10% in pre-primary institutions.

## Materials

### Lectures and Exercises

Minnie’s lecture “Understanding”, was aimed at developing the parents’ ability to understand their SEN children. The purpose of the exercise “Matches” was to show parents how to be aware of and correctly present information to a SEN child. Each parent had to come up with instructions to explain an action. Next, they changed places and independently had to present the material so that another parent understood it. The purpose of the exercise “Creativity” was to teach parents to offer adequate assistance to the child, focusing on the SEN child’s preferences. Each parent received a card with incomplete images. Under the instructions of the lecturer, parents finished drawing (flowers, animals, sun) these images. The “Action” exercise presented the idea of SEN children acquiring independent skills, creating a situation for parents to navigate on their own without guidance from psychologists.

### Questionnaires

#### Questionnaire No.1 “The parent child relationship self-report”

The questionnaire included 20 questions about how parents communicate with the child, whether they find a common language, what difficulties arise in the process of communication, and whether parents need the help of a teacher or a psychologist. The response format for the questionnaire was based on a 5-point Likert-type scale, ranging from 1 (strongly disagree) to 5 (strongly agree). Additionally, some sections of the questionnaire included Yes/No questions to capture definitive responses on specific aspects of the parent-child relationship.

#### Questionnaire No.2 “Identifying core parent-child relationship challenges”

The questionnaire included 130 questions where it was necessary to agree/disagree with the statements. This questionnaire was chosen to streamline the process of identifying specific relationship dynamics between parents and their SEN children. The questionnaire aimed to pinpoint specific relationship dynamics. The scoring system was binary, with ‘agree’ responses assigned a score of 1 and ‘disagree’ responses assigned a score of 0.

### Procedure

The program for accompanying parents with SEN children lasted seven days and was run in schools located in Almaty.

Questionnaire’s No.1 score was calculated by summing up the responses for each item. The higher the total score, the more positive the assessed aspect of the parent-child relationship showed. The scores from different subscales were analyzed both individually and collectively to gain a comprehensive understanding of the overall relationship quality.

Questionnaire’s No.2 score was calculated by summing up the points from each response. Initially, the raw responses from the FRA were gathered and a preliminary assessment was done to identify recurring themes and patterns.

The goals and the progress of each exercise were recorded. Also, each lecture was analysed with the help of a psychologist to find solutions for misunderstandings on behalf of the parents.

In the “Understanding” lecture, parents heard about how important it is to be aware of the behaviour of their child, including gestures and body language. The parents were exposed to the benefits of the correct choice of tone and intonation, as well as the importance of praise. Thanks to the exercise “Matches”, the parents were able to present information to their SEN child. During the exercise “Creativity”, the parents followed the given recommendations well, which indicated their ability to effectively adapt to the child’s creative needs and preferences. During the exercise “Action”, the parents answered questions about how often they do the work instead of the child and the child’s reaction to doing the task on their own.

Initially, the educators and social scientists, talked about the specific features of the emotional development of children of different ages. The psychologist, in turn, given information about the aggression that occurs in the child, explained their causes and how to deal with them. The next day, together with psychologists, an operational analysis and discussion were carried out on how parents communicated with children using a fairy tale and what other problems arose during communication. It all ended with conclusions about the effectiveness of using this technique in the future.

## Results

The main problems faced by parents and teachers in their interaction with SEN children were identified by using Questionnaire No.2 “Identification of the main problems in the relationship between parents and children” (Table 2). The information presented in Table 2 were derived from a combination of observational data, detailed analysis of parent-child interactions, and feedback from structured exercises and lectures.

**Table 2:** Summary of parental training exercises and methods.


EXERCISE/METHOD	DESCRIPTION/GOAL	KEY POINTS/OUTCOMES

Attention	Place parents in the child’s perspective during discussions	Understand child’s self-esteem and how often parents discuss the child with others

Activity	Reproduce child’s movements	Enhance sensory experience, highlight difficulties faced by parents

Dialogue or monologue	Identify parent communication styles	Types: 1) Parents-dictators 2) Custodial parents 3) Parents-diplomats

Fairy tale Activity	Use fairy tales to solve motivational problems	Importance of teacher’s skills; parents identify child problems and relate them to fairy tales


The relationship between parents and children was presented in percentages. It was determined that 56% of parents don’t support their child, 34% don’t know how to influence their child, and only 10% of parents understand their children.

Analyzing the responses revealed key indicators in parental attitudes, such as hyper protection, hypo protection, indulgence, and inconsistent upbringing styles (Table 3). Higher scores indicated a greater presence of the respective attitude being measured, such as hyper protection or indulgence. For example, a high total score in the hyper protection segment suggested a strong tendency towards overprotective behaviour. The insights from this test informed the creation of targeted courses and training sessions for parents at the school, ensuring they address the core challenges identified.

**Table 3:** Results of the survey “Analysis of family relationships”.


RESULTS	PERCENTAGE	DESCRIPTION

Instability of Parenting Style	15%	Constant changes in the style of education, lack of gradation in education.

Hyper protection	13%	Parents pay too much attention to the upbringing of their child.

Indulgence	13%	Over-indulging a child in various things at his will.

Excessive requirements	12%	High demands on the child in the field of development, education, talent and sociology.

Ignoring	9%	Parents lack the strength and desire to meet the needs of the child.

Excessive demands-prohibitions	8%	Many prohibitions on various activities, communication, thoughts on the part of parents.

Insufficiency of requirements-prohibitions	8%	The absence of any prohibitions for the child, that is, all permissibility.

Minimality of sanctions	7%	Lack of sanctions and punishments for non-performance of work.

Lack of child responsibilities	6%	Parents give a small number of responsibilities to children in the home.

Hypo protection	5%	Parents do not have time for the development and upbringing of their child, respectively, do not deal with the child at all.

Excessive sanctions	4%	Severe penalties for non-compliance with assigned tasks.


The purpose of the lecture and exercises was to increase the level of understanding and empathy of parents as well as to lower a parent’s expectations and high goals for the child. Thus, the skills of independent work of children without the help of parents was noticed.

When using the program for the study of psychological and pedagogical competencies, holding meetings between teachers and parents, the parents gained new knowledge that could protect SEN children from the progression of diseases at the psychological level. These educational sessions played a pivotal role in enhancing parents’ psychological and pedagogical competencies. During these interactions, teachers, and social workers imparted crucial information about the nuanced emotional development stages children undergo at different ages. Parents responded overwhelmingly positive, with many expressing gratitude for the insights into the triggers of aggression in children, and strategies to address them. The meetings delved into the potential risks of neglecting emotional well-being, such as the progression of mental and emotional disorders in children. Additionally, the program introduced parents to reflective games, proven tools to address a child’s fears creatively without exerting undue psychological stress. Similarly, seminars exploring these game types were also conducted. Parents left these sessions better equipped and more confident in supporting their children’s emotional and psychological needs.

Similarly, narratives that depicted parents’ reluctance to set boundaries or say ‘no’ to their children were instrumental in recognizing indulgence as a key indicator. Observations further enriched this process. By watching interactions between parents and their children during workshops or game trainings, behaviours emblematic of certain indicators were noted. For example, inconsistency in upbringing style was observed when parents exhibited erratic behaviour – being overly strict at one moment and then overly permissive the next. It is important to note that those parents (45%) who had difficulties had to repeat the conversation with their children again.

## Discussion

Inclusive education is at the forefront of educational research, with a significant emphasis on the interplay between teachers, psychologists, and parents in the development of SEN children. The current study brings into focus the pivotal role of parents and the unique challenges faced by them.

Exploring global perspectives on inclusive education and parental support, various strategies and approaches can be observed in different countries. In the Ukraine, for instance, there is an emphasis on creating specialized training programs for parents to better understand and support their children’s unique needs, as echoed by Kutsyn (2019). China, on the other hand, has made significant strides in integrating technology into their inclusive education strategies (Dor, 2022). They offer online resources and platforms where parents can collaborate with educators and therapists, ensuring a cohesive approach to address children’s needs. Interestingly in France, the emphasis is on community involvement (Selvachandran et al., 2022). That is, local community groups and organizations play a pivotal role in organizing workshops and awareness campaigns, ensuring that parents are not just informed but are also part of a larger supportive network. These varied approaches, from Ukraine’s training programs to China’s technological integration and France’s community-centric strategy, highlight the diverse ways in which countries are tackling the challenges faced by parents of SEN children. The overarching theme across these nations remains consistent: the importance of equipping parents with the right tools, knowledge, and support networks to foster a conducive learning environment for their children.

The research by Sapargalieva, Aralbayeva and Resbekov (2015) delves into the nuances of professional training for individuals in helping professions. The study is methodically structured as a formative experiment, capturing comprehensive data. This work emphasizes three critical pedagogical conditions crucial for molding professional competence: specialization, communication, and personal development.

Research by Miakushko (2019) delineates the importance of understanding the dynamics in relationships when supporting children with intellectual disabilities. These insights are congruent with findings from the current study which accentuate the significance of nurturing relationships between school psychologists, teachers and parents. The need for this synergy is further supported by Mann and Gilmore (2021), who point out potential barriers in forming constructive parent-teacher partnerships, thereby emphasizing the necessity for more integrated efforts in inclusive educational contexts.

The program proposed in the current study to mediate the dynamics between parents, psychologists, and teachers is reflected in findings by Pihlainen et al. (2022). Further, the focus on parental competencies during the unique challenges posed by the COVID-19 lockdown aligns with the objectives of the program designed in this study. Osario-Saez et al. (2021) discuss parents’ acceptance of educational technology, the broader theme of equipping parents with the necessary tools and knowledge, plus technological or psychological, aligns with themes from the current study.

Findings by Hoferichter et al. (2021) on the varying impacts of support from different sources on child well-being complement the focus of the present study, while research by Caldarera et al. (2021) on support groups emphasizes the universal need for parental understanding and support. Moreover, research from Butler et al. (2022) and Attard and Booth (2023) reinforces the sentiment of this study concerning the importance of strengthening family ties and creating a holistic environment for children.

Research endeavours such as those by Chechko et al. (2022), Curra et al. (2023), and Dor (2022) emphasize the need for early intervention, collaboration, and comprehensive approaches in inclusive education, themes which resonate with the current study’s objectives. Studies like those by Mukashev and Somerton (2023) offer a global perspective to inclusive education practices, providing a broader context to the domain. Furthermore, research from Zhasulan et al. (2022), Selvachandran et al. (2022) highlight the multi-dimensional challenges and avenues in inclusive education.

To summarize, one of the main psychological and pedagogical conditions is the use and development of methods, courses and training programs that will be openly available to parents who will have the opportunity to constantly develop their communication with other parents’ children. In addition, it is important to have the right specialist, an assistant psychologist, and teacher, who will constantly analyse the activities and point out the positive aspects and mistakes. Of course, the presence of practice, the development of understanding and emotional stability are the criteria that not only teachers and psychologists should have, but also parents.

The methods employed in this study, including the FRA and the program of exercises and lectures, were meticulously chosen to delve deep into the intricate dynamics of parent-child relationships, especially when the child has special educational needs. Drawing from existing research and the distinct experiences of parents, these methods aimed to unearth the primary challenges parents face in communication and understanding. The focus was not just on identifying problems, but also on equipping parents with the necessary skills and empathy to bridge the communication gap and foster stronger relationships with their children.

The results of this study showed a positive impact on parents and their pedagogical and psychological activities. Also, this program is recommended for accompanying parents with SEN children, keeping in mind the number of classes can be adapted according to the performance of parents. Significantly, it proves essential whilst adopting a program to accompany parent with SEN children to work individually with each participant in the process. In particular, the main task endeavours to teach parents the ability to understand their children who need special conditions for development. Also, by analysing and summarizing the information received, it was advisable to create a program that included the introduction of classes, exercises and lectures that would develop precise competencies in parents and solve problems parents encountered. For example, through the exercises “Action”, “Emotions”, and “Sculptor”, parents were able to experience the emotions their children undergo when children do not understand or were afraid.

## Conclusion

The realm of inclusive education is complex, continually evolving, and rife with challenges. This study, in its essence, endeavoured to address one of the primary stakeholders in this situation: parents of SEN children. It identified that at the heart of many challenges faced by these parents lies communication and understanding. By recognizing this, the study made strides in introducing a structured program aimed at equipping parents with the necessary tools, insights, and confidence to better engage with their children. Drawing upon methods such as FRA and a combination of lectures and exercises, the research provided a holistic approach to addressing these challenges. A noteworthy aspect of this research was its emphasis on strengthening the bond between parents and educational professionals, notably school psychologists and teachers. The establishment of mutual trust and the creation of a conducive psychological environment stood out as pivotal in ensuring the program’s success.

The study’s recommendation to consider the influence of peers and friends in future research underlines the multifaceted nature of inclusive education. While parents are crucial, the broader social environment of a child also plays a significant role in their development. One pivotal takeaway from the research is the need for continuous development. As the educational landscape evolves, so do the challenges. Thus, ensuring that parents have access to open, flexible training modules becomes paramount. Coupled with the guidance of trained specialists, like psychologists or teachers, this continuous learning can lead to better communication, deeper understanding, and more effective inclusion.

Additionally, the emphasis on individualized attention in the program speaks volumes about the uniqueness of each parent-child relationship. No two experiences are identical, and catering to these nuances is what makes interventions like the proposed program stand out. In closing, the study provides a robust framework for addressing the challenges faced by parents of SEN children. By focusing on enhancing communication, fostering trust, and promoting continuous learning, it paves the way for more inclusive, understanding, and supportive educational environments. The journey of inclusive education is long and intricate, and this research serves as a beacon, guiding stakeholders through some of its most challenging terrains.
